# Implementation of a multidisciplinary discharge videoconference for children with medical complexity: a pilot study

**DOI:** 10.1186/s40814-020-00572-7

**Published:** 2020-02-18

**Authors:** Noga L. Ravid, Kayla Zamora, Roberta Rehm, Megumi Okumura, John Takayama, Sunitha Kaiser

**Affiliations:** 1 Department of Pediatrics, University of California, San Francisco, 550 16th St. 5th floor, Box 0110, San Francisco, CA 94143 USA; 2 San Francisco School of Medicine, University of California, 513 Parnassus Avenue, San Francisco, CA 94143 USA; 3 Department of Family Health Care Nursing, University of California at San Francisco, 2 Koret Way, San Francisco, CA 94143 USA

**Keywords:** Children with medical complexity, Hospital to home transition, Telemedicine

## Abstract

**Background:**

The hospital to home transition for children with medical complexity (CMC) poses many challenges, including suboptimal communication between the hospital and medical home. Our objective was to evaluate the implementation of a discharge videoconference incorporating the patient, caregiver, primary care provider (PCP), hospitalist physician, and case manager.

**Methods:**

We evaluated implementation of this pilot intervention at a freestanding tertiary care children’s hospital using mixed methods. A discharge videoconference was conducted for hospitalized children (< 18 years old) meeting complex chronic disease (C-CD) criteria. We collected field notes and conducted surveys and semi-structured interviews. Outcomes included adoption, cost, acceptability, feasibility, and appropriateness. Adoption, cost, and acceptability were analyzed using descriptive statistics. Acceptability, feasibility, and appropriateness were summarized using thematic content analysis.

**Results:**

*Adoption*: A total of 4 CMC (9% of the 44 eligible children) had discharge videoconferences conducted. *Cost* (*in provider time*): On average, videoconferences took 5 min to schedule and lasted 21.5 min. *Acceptability*: All hospitalists involved (*n* = 4) were very likely to participate again. Interviews with caregivers (*n* = 4) and PCPs (*n* = 5) demonstrated that for those participating, videoconferences were acceptable and appropriate due to benefits including development of a shared understanding, remote physical assessment by the PCP, transparency, and humanization of the care handoff, and increased PCP comfort with care of CMC. *Feasibility:* Barriers included internet connection quality and scheduling constraints.

**Conclusions:**

This novel, visual approach to discharge communication for CMC had low adoption, possibly related to recruitment strategy. The videoconference posed low time burdens, and participating physicians and caregivers found them acceptable due to a variety of benefits. We identified several feasibility barriers that could be targeted in future implementation efforts.

## Introduction

Children with medical complexity (CMC) are a growing population of children in the USA. CMC are high utilizers of health of care [1] and undergo frequent care transitions (e.g., hospital to home) [2, 3]. Hospitalists play an important role in care for CMC, including discharge from the hospital [4]. Research has shown that the hospital to home transition is fraught with obstacles, including insufficient and ineffective communication [5–7].

Prior work by Solan et al. described numerous problems with hospitalist-primary care provider (PCP) communication at the time of hospital discharge, including perceived devaluation of the PCP and unclear post-discharge responsibilities, and identified videoconferences as a potential solution [8]. Multidisciplinary discharge videoconferences have been shown to improve communication, increase access to hospital staff and information, and decrease medication errors during geriatric hospital to post-acute care transitions [9]. However, this approach has not been studied in children. Our objective was to evaluate implementation of a multidisciplinary discharge videoconference in order to inform future efforts to improve hospital to home transitions for CMC.

## Methods

### Study design and population

We evaluated the implementation of this pilot intervention using mixed methods. Our study population included English- and Spanish-speaking caregivers and healthcare providers of CMC. CMC (< 18 years old) were eligible for the intervention if they were hospitalized for ≥ 3 days at the study site (a freestanding tertiary-care children’s hospital) from August 2016 to February 2017. The end of the pilot recruitment period was determined by availability of allocated research staff time. CMC were identified using complex chronic disease (C-CD) criteria [10]: technology dependence ≥ 6 months, or a significant medical condition affecting ≥ 2 body systems for > 1 year. We identified CMC via email survey of on-service hospitalists once every 2 weeks during the study period. This strategy was selected based on available resources and personnel. We also publicized the study at monthly hospitalist group meetings three times during the study interval. Caregivers of eligible CMC were approached to participate in the study (convenience sampling). The study was approved by the institutional review board.

**Fig. 1:**
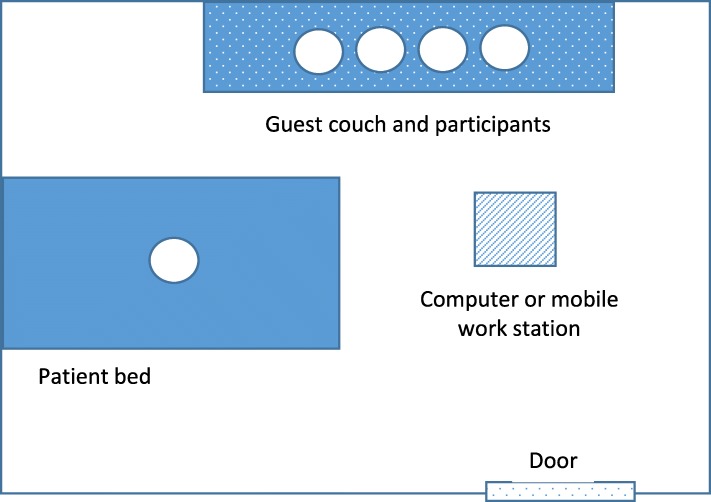
Diagram of videoconference setup

Approximately 3 days prior to planned hospital discharge, research staff contacted PCPs, hospitalists, and case managers to schedule the discharge videoconference. Appointment time for the conference was determined by first assessing PCP availability and then coordinating hospitalist and case manager availability. Initial PCP contact was made via telephone at PCP office, and subsequent communication was conducted via direct phone line, cell phone, or email as directed by PCP. Hospitalist and case manager contact was made via email, text message, or in person. The videoconference was conducted in the patient’s private room on a laptop via a secure, HIPAA compliant videoconference platform, Webex (Version 2.6.1.39, Santa Clara, CA). The patient, caregiver, inpatient case manager, hospitalist, PCP, research team member, and public health nurse (if applicable) were the invited participants. PCPs and public health nurses joined the conference remotely (Table 1). All others were present in the patient’s room and visible on the videoconference (Fig. 1). The videoconference covered topics specified in a nationally recognized framework of pediatric discharge, including details on the hospital course, changes in the child’s medications, and outpatient needs for the PCP to follow up on (e.g., clinical assessment, labs, outpatient appointments, nutrition, outpatient management, and contingency plans) [11]. While these topics were already considered standard of care for discharge communication at our institution, the novel aspect of this intervention lies in its use of a videoconference including the patient and guardian, rather than provider-to-provider telephone call.

**Table 1:** Videoconference preparation and setup

Participants	▪ Patient and guardian ▪ Hospitalist ▪ PCP ▪ Case manager ▪ Home or public health nurse ▪ Interpreter
Hardware	▪ Hospital: laptop or mobile workstation with broadband internet, video, and audio capabilities ▪ PCP: computer or mobile device with broadband internet, video, and audio capabilities
Preparation	▪ 1–2 days prior, coordinator emails hospitalist and PCP Webex invitation and link to videoconference ▪ Hospital computers have Webex app pre-installed ▪ PCP installs Webex app on mobile or desktop device ahead of videoconference ▪ Hospitalist is responsible for starting videoconference via provided link
Positioning	▪ Patient in bed or in guardian lap ▪ Guardian, hospitalist, case manager, and interpreter (if present) side-by-side, facing camera/PCP ▪ If patient is in bed, camera is rotated to show patient at appropriate moments

### Data collection

We collected field notes on time spent in scheduling and conducting conferences. We conducted electronic surveys of hospitalists and caregivers to collect data on overall satisfaction and demographics, respectively. After the first PCP follow-up outpatient visit (~ 1 week post-discharge), we conducted semi-structured telephone interviews with PCPs and caregivers, using a telephone Spanish interpreter when appropriate. The research team developed interview guides and piloted these with caregivers and PCPs to ensure clarity and face validity of questions (Additional file 1). Interviews lasted 20–30 min and were audio-recorded, professionally transcribed, de-identified, and reviewed for accuracy.

### Outcomes

We determined implementation outcomes including *adoption* (intention to try the intervention), *cost* (in provider time), *acceptability* (perception that the intervention is satisfactory), *feasibility* (the extent to which the intervention could be successfully carried out), and *appropriateness* (perceived fit, relevance, or compatibility) [12].

### Analysis

Quantitative data on adoption, cost, and acceptability were summarized using descriptive statistics. Qualitative data on acceptability, feasibility, and appropriateness were analyzed using thematic content analysis [13]. The first stage of coding involved reading through all collected data to gain an overall understanding of participant responses. Preliminary codes were created inductively by two independent coders. Codes were then synthesized into themes in discussion with the entire study group. Qualitative data were analyzed using Dedoose (version 8.0.42, Hermosa Beach, CA).

## Results

### Adoption

**Fig. 2:**
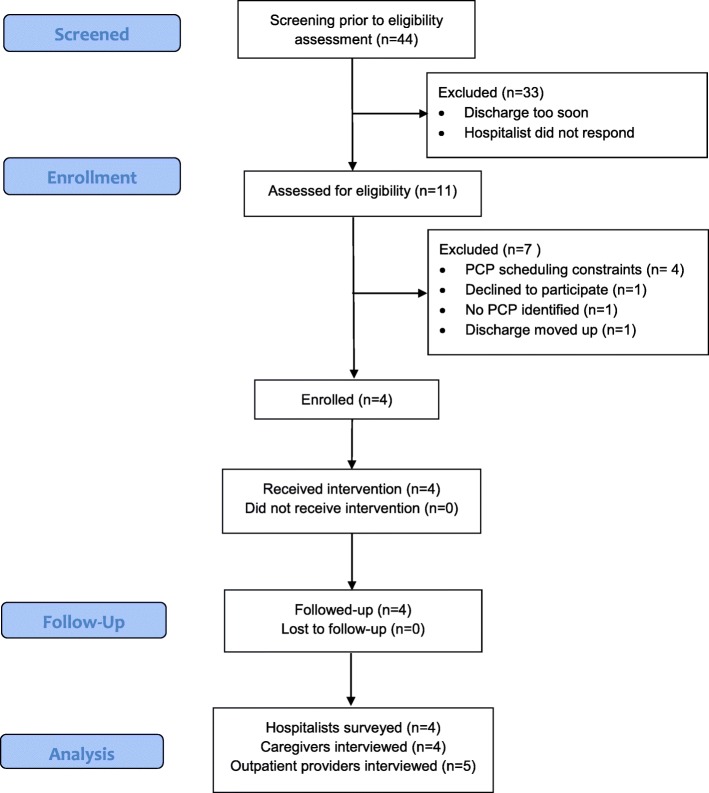
CONSORT diagram

Email surveys were sent to hospitalists every 2 weeks during the study period to identify eligible patients, and 44 eligible patients were identified. Of these, 25% (*n* = 11) were approached. The others were not approached because anticipated discharge was too soon (< 2 days) to arrange the videoconference, or the attending hospitalist did not respond regarding approaching the patient despite two contact attempts. Of the patients approached, 64% (*n* = 7) did not participate for the following reasons: PCP scheduling constraints (*n* = 4); parents did not feel conference would be valuable (*n* = 1); discharge timing changed, leaving inadequate time to reschedule (*n* = 1); and no PCP identified (*n* = 1). A total of 4 children (36% of those approached, 9% of eligible) received the intervention. Details of recruitment are shown in Fig. 2 below [14].

### Cost

Cost was quantified in medical provider time. On average, videoconferences took 5 min and 1.75 contact attempts to schedule and lasted 21.5 min. The videoconferences were primarily scheduled by phone and held in the afternoons (Table 2).

**Table 2:** Cost in provider time

Number of scheduling encounters	Modality of scheduling encounters	Total time scheduling	Videoconference duration	Videoconference time
1	Phone	10	12 min	1600
1	Phone	5	16 min	1500
1	Phone	5	20 min	0900
4	Phone and email	15	38 min*	1340

*15 min for technologic difficulties

### Acceptability, feasibility, and appropriateness

All four hospitalists who participated in the videoconferences were surveyed using the Likert scales. In each case, the hospitalist surveyed was attending on the day of discharge, and in some cases, s/he was also the referring hospitalist. All hospitalists surveyed reported that they were very likely to participate in future discharge videoconferences, as well as recommend participation to a colleague.

A total of four caregivers and five outpatient healthcare providers (three physicians, one nurse practitioner, one public health nurse) were interviewed. Caregivers were all mothers and had diverse educational attainment (Table 3). We identified several common themes in our process evaluation (Table 4).

**Table 3:** Participant characteristics

Age of child, *y*, no. (%)	
< 1	2 (50)
1–5	2 (50)
Sex of child, no. (%)	
M	2 (50)
F	2 (50)
Race and/or ethnicity of child, no. (%)	
Black	1 (25)
White	4 (25)
Hispanic	2 (50)
Other	1 (25)
Relationship of caregiver to child, no. (%)	
Mother	4 (100)
Age of caregiver, *y*, no. (%)	
18–24	1 (25)
25–34	3 (75)
Caregiver education, no. (%)	
Some high school	1 (25)
High school graduate or GED	1 (25)
Some college or 2-year degree	1 (25)
4-year college degree	1 (25)
Preferred caregiver language, no. (%)	
English	3 (75)
Spanish	1 (25)
Type of outpatient provider, no. (%)	
Nurse practitioner	1 (20)
Physician	3 (60)
Public health nurse	1 (20)
Outpatient provider practice setting, no. (%)	
FQHC	1 (20)
Private practice	1 (20)
Indian health service	1 (20)
County health service	2 (40)

**Table 4:** Themes and illustrative quotes regarding the use of a discharge videoconference for CMC

Theme	Illustrative quote from PCP	Illustrative quote from caregiver
Development of shared understanding	“I liked it because I think it kind of empowers [caregivers] to advocate too and to ask questions or concerns, kind of like when you do rounds and involve the family.”	“Less of me having to call one person and me explain what had happened there at the hospital…and there’s just a lot of terms and different medical stuff that I probably wouldn’t have been able to explain to [PCP].”
Benefits of remote physical assessment		“If [Patient] were awake [visual aspect] would have been valuable…would have been helpful to [PCP] so he knows how [Patient] acts on a normal basis.”
Transparency	“I think mom was able to see the communication between providers…with the mom there, so that she hears that and hears it from both sides.”	
Humanizing the handoff of care	“Well I think on a kind of subjective level, it is just kind of nice to have a face to the [caregiver] and get a sense of how she was doing and to see her sort of nodding as people were talking and stuff like that, I think is just helpful to know that they are kind of on board with what is going on.”	“Sitting and talking with [PCP]…he wasn’t freaking out, so it just made me feel like, “Oh, I don’t need to freak out”…so it actually calmed down my nerves.”
Increasing PCP comfort with care of CMC	“I felt so much more comfortable when I saw [Patient] on my schedule knowing that, okay I know *exactly* what happened down there and I know exactly what to look for.”	
Feasibility barriers	“I just feel like it was a little tiny bit confusing because I couldn’t really tell who was calling … if we would have been able to see all of their faces and that way we could have known who was speaking at the moment—then that would have been better.”	“Aside from reimbursement, just providers being so busy…I could conceive that some providers who are a little bit overwhelmed could just say this is one more thing I don’t want to deal with.”

### Development of a shared understanding

Both caregivers and PCPs appreciated having the same information and developing a shared vision of the care plan. For caregivers, the shared understanding also provided reassurance.

We are all on the same page, and we all know what is expected of what I’m supposed to know and we don’t get lost in communication. (PCP)

When we came for our follow-up appointment with [PCP], he remembered everything we had discussed…I was able to go into the appointment knowing that, okay, all my concerns have been addressed…we all knew what we were going to do. (Caregiver)

### Benefits of remote physical assessment for PCP

Visualizing the patient provided important clinical information for PCPs:

I think if I had heard the stridor down in the office, I would have been like ‘holy cow’... so to hear…over the teleconference, and then hear it less when I saw her in the office, was so reassuring…and helpful. (PCP)

I liked being able to see the baby and she looked absolutely more comfortable than when I had seen her before. (PCP)

### Transparency

Transparency about both the act of sharing information and the detailed discussion was valued by both PCPs and caregivers. Awareness of each other’s knowledge, in addition to the information itself, was important.

The parents knew already coming in what I knew… and they knew I knew. (PCP)

Usually they send an email or try to call his doctor, and I wouldn’t know exactly what was being said…so with [conference] I know what is being said about [the patient’s] care. (Caregiver)

### Humanizing the handoff of care

Caregivers and providers endorsed a comfort derived from seeing one another through video. This relational aspect facilitated conversation and strengthened bonds among all parties.

It was just very nice to be able to put a face with the family, and a face with [Hospitalist]…It felt like we were able to have a little more back and forth conversation…I can’t think of a specific reason other than just my gut was like, this is really nice, and it felt good. (PCP)

[PCP] was a familiar face in a sea of craziness. So, it was comforting... Just seeing [PCP] was like, “Oh my God. What an event. And so, it was therapeutic in a way. (Caregiver)

### Increasing PCP comfort with care of CMC

Providers noted that the videoconference increased their level of comfort in assuming care of the patient.

The thing that makes me most comfortable is having had a discussion… I don’t see a ton of kids with all these various chronic and complex diseases. And so, while they’re familiar to me, this is not something that’s every day, so I don’t have the expertise to confidently make a definitive decision about what the next step is all the time. (PCP)

### Feasibility barriers

Technological, financial, and time constraints posed barriers to conducting the discharge videoconference. The primary technological barrier was quality of internet access in PCP offices. Financial and time constraints were intertwined, related to the logistics of scheduling time for an important but non-billable activity.

I think just our bandwidth wasn’t—our internet wasn’t so good and reception kept going in or out. (PCP)

It is a huge chunk of time out of our day when we have patients that we want to be seeing and we can’t even bill for [the discharge videoconference]…I think ideally if you are going to do it, you would want to be able to have it scheduled [and billed] just like a patient. (PCP)

## Discussion

In this pilot study, we found that a multidisciplinary discharge videoconference for CMC was acceptable for those participating due to benefits including development of a shared understanding of the patient’s care plan, remote physical assessment by the PCP, transparency, humanization of the care handoff, and increased PCP comfort with care of CMC. However, the videoconferences had low adoption. In addition, feasibility barriers included internet access and time constraints.

Adoption of this intervention was concerningly low, with only 9% of those eligible and 36% of those approached participating. The major barrier to adoption was timely identification and contact with eligible children/families. Hospitalists were informed of the study details and monthly goals and contacted every 2 weeks via email regarding recruitment. However, when contacted, barriers included (1) timing too close to discharge to arrange the videoconference and (2) permission for approaching the child/family was not obtained in a timely manner. These barriers may have been heightened by the competing priorities faced by hospitalists including clinical duties, supervisory responsibilities, and administrative/non-clinical tasks. Potential strategies for improving adoption include more frequent eligibility screening, in-person discussion of recruitment at daily discharge planning rounds, discharge bundles to improve prediction of discharge timing [15], or opt-out formats with standardized scheduling of discharge videoconferences for all CMC.

We identified several feasibility barriers, reflecting the challenges of implementing complex care interventions in healthcare settings. Technological barriers with internet access may improve as infrastructure for broadband internet access becomes more available, including in rural areas [16]. Scheduling and related financial obstacles may be mitigated as models of payment for CMC move away from fee-for-service towards value-based or bundled payments, which encompass care coordination and case management [17]. In addition, reimbursement for telehealth case management and patient care may provide a precedent for billing [18].

If future implementation efforts are able to increase adoption and address feasibility barriers, this family-centered intervention may help achieve proposed goals around improving care coordination and self-efficacy in the hospital to home transition for CMC [4, 7, 19]. Discharge videoconferences may also support the comfort and confidence of the PCP caring for CMC, a barrier identified in prior studies [20, 21].

While teleconferences are not new to CMC, to our knowledge, this is the first study of a multidisciplinary discharge videoconference for children. Our findings are consistent with a previous study utilizing a multidisciplinary videoconference during geriatric hospital to post-acute care transition, which demonstrated improvements in communication, post-acute provider access to hospital staff, and medication errors [9]. Our intervention differed in its inclusion of the patient and caregiver in the handoff, a practice in keeping with principles of family-centered care [22]. Two large pediatric RCTs evaluating the impact of post-discharge contact with patients and families found mixed effects on 30-day reutilization [23, 24]. However, our intervention differed both in its pre-discharge timing and incorporation of the PCP, both of which, we found to have potentially broader effects, such as increasing PCP comfort.

There were several limitations to our analysis. This was a small pilot study in a single academic institution, and results may not be generalizable to other settings. For example, institutional infrastructure such as equipment and software for telemedicine was established, accessible, and free at the time of this study. We did not evaluate the costs of such equipment or parent time costs in this analysis. In addition, arrangement of videoconferences was managed by a hospitalist with extensive knowledge of inpatient workflows and time dedicated for this purpose. The small sample size is a significant limitation of this study, and the recruitment strategy may have been a contributing factor. Future qualitative work with hospitalists, non-participating PCPs, and/or non-participating parents may yield insights and potential solutions to improve recruitment efforts. However, our results describe innovative solutions to challenges in the hospital to home transition for CMC that merit further exploration. Future study should involve larger-scale implementation to see if our findings are replicable and generalizable. In addition, future work should explore whether discharge videoconferences can improve quality of care and patient outcomes.

## Conclusions

As pediatric health systems seek to improve discharge of CMC, developing mechanisms to ensure continuity of care and to meet the needs of families is critical. This visual approach to discharge communication for CMC had low adoption, possibly related to recruitment strategy. The videoconference posed low time burdens, and participating physicians and caregivers found them acceptable due to a variety of benefits. We identified several adoption and feasibility barriers that could be targeted in future implementation efforts.

## Supplementary information

**Additional file 1.** Interview Guide.

## Data Availability

The datasets generated and analyzed during the current study are available from the corresponding author on reasonable request.

## Abbreviations

C-CD: Chronic complex disease
CMC: Children with medical complexity
PCP: Primary care provider
