# Supplementary material for: Implementation of a multidisciplinary discharge videoconference for children with medical complexity: a pilot study
Source: Pilot Feasibility Stud. 2020 Feb 18;6:27. doi: 10.1186/s40814-020-00572-7 (PMC7027051; doi:10.1186/s40814-020-00572-7)
Supplement: Supplementary file 1 — Additional file 1. Interview Guide. [file 40814_2020_572_MOESM1_ESM.docx]

Interview Guide

**Primary Care Provider Participant**

“I’d like to start with general questions about your experiences with discharge for medically complex children, and then after talk about the intervention itself.”

| **Questions** | **Prompts** |
| --- | --- |
| Can you tell me about your experiences generally with discharge for medically complex kids? | - What were the best parts about previous discharges? - What has been bad about previous discharges? |
| How did X child’s recent discharge compare with past experience? | - What was good about it? - What was less optimal? |
| What would your practice ideally want for discharge information and handoff for a medically complex child? | - What information components would be valuable to you? - What types of patients would this meeting be most helpful for? |
| What would make you more comfortable assuming care of a medically complex child after discharge? |  |
| Tell me what you thought about the discharge teleconference? | - How did it impact quality of transfer? - How did the teleconference help you as compared with written summaries or phone calls? - Compared with past discharges? - Was the visual aspect helpful? - What were your thoughts/feelings on having case management present? - What were your thoughts/feelings on having family present? |
| If we made this teleconference standard practice for CMC discharge: | - Would you use it? - If yes, why? What value added? - If no, why? - How would you schedule this? - What barriers do you anticipate? - How does reimbursement impact your capacity to participate? - What suggestions do you have? |

Interview Guide

**Parent /Guardian Participant**

“I’d like to start with general questions about your experiences with, and then after talk about the intervention itself.”

| **Questions** | **Prompts** |
| --- | --- |
| Tell me a little about past hospital admissions and discharges/transitions to home. |  |
| Can you tell me about your experience with your child’s recent discharge/transition to home? | - Tell me about when you first found out about your home planning - What was that process like? - Who did the home planning with you? - What home planning activities include? - What was good or bad about how the transfer was done? - How did it compare with past discharges? - Can you give me an example? |
| In general, what makes you comfortable when leaving the hospital with your child? | - Have there been issues where you’re uncomfortable going home? Why? |
| Tell me what you thought about the discharge teleconference? | - How is or isn’t the visual aspect valuable? - Are there any changes you would suggest in how the conference was organized or carried out? |
| How, if at all, did the teleconference affect your ability to care for your child following hospital discharge? | - How does that compare with past discharges? |
| If we made this teleconference standard practice for your child’s discharge: | - Would you want to take part in it? - If yes, what does it add? - If not, why? |
